# Results of the observational prospective RealFLOT study

**DOI:** 10.1186/s12885-021-08768-7

**Published:** 2021-10-08

**Authors:** Elisa Giommoni, Daniele Lavacchi, Giuseppe Tirino, Lorenzo Fornaro, Francesco Iachetta, Carmelo Pozzo, Maria Antonietta Satolli, Andrea Spallanzani, Marco Puzzoni, Silvia Stragliotto, Michele Sisani, Vincenzo Formica, Filippo Giovanardi, Antonia Strippoli, Michele Prisciandaro, Samantha Di Donato, Luca Pompella, Irene Pecora, Alessandra Romagnani, Sara Fancelli, Marco Brugia, Serena Pillozzi, Ferdinando De Vita, Lorenzo Antonuzzo

**Affiliations:** 1 Medical Oncology Unit, AOU Careggi, Florence, Italy; 2 Division of Medical Oncology, Department of Precision Medicine, University of Study of Campania “L. Vanvitelli”, Naples, Italy; 3 Medical Oncology, Azienda Ospedaliero-Universitaria Pisana, Pisa, Italy; 4 Medical Oncology Unit, Clinical Cancer Center, AUSL-IRCCS, Reggio Emilia, Italy; 5 Medical Oncology, Fondazione Policlinico Universitario “A. Gemelli”, IRCCS, Rome, Italy; 6 Department of Medical Oncology, University of Turin, Turin, Italy; 7 University Hospital of Modena, Modena, Italy; 8 Medical Oncology Department, University Hospital, University of Cagliari, Cagliari, Italy; 9 Oncology Unit - Dipartimento di Oncologia Clinica e Sperimentale Istituto Oncologico Veneto IOV-IRCCS, Padua, Italy; 10 Medical Oncology, Usl Toscana Sud est, Arezzo, Italy; 11 Internal Medicine Department “Tor Vergata” University Hospital, Rome, Italy; 12 Medical Oncology, Azienda Unità Sanitaria Locale-IRCCS di Reggio Emilia, Reggio Emilia, Italy; 13 Medical Oncology Department, Fondazione IRCCS Istituto Nazionale dei Tumori, Milan, Italy; 14 Medical Oncology, Department Nuovo Ospedale-Santo Stefano Istituto Toscano Tumori, Prato, Italy; 15 Department of Experimental and Clinical Medicine, University of Firenze, Florence, Italy; 16 Clinical Oncology Unit, AOU Careggi, Largo Brambilla 3, 50134 Florence, Italy

**Keywords:** FLOT, Perioperative chemotherapy, Gastric cancer, Gastro-oesophageal junction adenocarcinoma, pCR, MSI

## Abstract

**Background:**

Perioperative FLOT (5-fluorouracil, oxaliplatin and docetaxel) has recently become the gold standard treatment for fit patients with operable gastric (GC) or gastroesophageal (GEJ) adenocarcinoma, getting a 5-year overall survival (OS) of 45%, over 23% with surgery alone.

**Methods:**

RealFLOT is an Italian, multicentric, observational trial, collecting data from patients with resectable GC or GEJ adenocarcinoma treated with perioperative FLOT. Aim of the study was to describe feasibility and safety of FLOT, pathological complete response rate (pCR), surgical outcomes and overall response rate (ORR) in an unselected real-world population. Additional analyses evaluated the correlation between pCR and survival and the prognostic role of microsatellite instability (MSI) status.

**Results:**

Of 206 patients enrolled that received perioperative FLOT at 15 Italian centers, 124 (60.2%) received at least 4 full-dose cycles, 190 (92.2%) underwent surgery, and 142 (68.9%) started the postoperative phase. Among patients who started the postoperative phase, 105 (51.0%) received FLOT, while 37 (18%) received de-intensified regimens, depending on clinical condition or previous toxicities. pCR was achieved in 7.3% of cases. Safety profile was consistent with literature. Neutropenia was the most common G 3–4 adverse event (AE): 19.9% in the preoperative phase and 16.9% in the postoperative phase. No toxic death was observed and 30-day postoperative mortality rate was 1.0%. ORR was 45.6% and disease control rate (DCR) was 94.2%. Disease-free survival (DFS) and OS were significantly longer in case of pCR (*p* = 0.009 and *p* = 0.023, respectively). A trend towards better DFS was observed among MSI-H patients.

**Conclusions:**

These real-world data confirm the feasibility of FLOT in an unselected population, representative of the clinical practice. pCR rate was lower than expected, nevertheless we confirm pCR as a predictive parameter of survival. In addition, MSI-H status seems to be a positive prognostic marker also in patients treated with taxane-containing triplets.

**Supplementary Information:**

The online version contains supplementary material available at 10.1186/s12885-021-08768-7.

## Background

Gastric cancer (GC) is the fifth most common cancer and the third most common cause of cancer mortality worldwide, with an estimated 783,000 deaths in 2018 [1]. Efforts to improve the outcome of GC remain a relevant medical issue.

Over the last 15 years, major advances in the multimodal treatment for GC, due to multiple phase III studies showing survival benefits for several perioperative chemotherapies (CTs) and adjuvant treatment strategies, have changed clinical management of the disease. Perioperative CT became a standard of care in Europe for resectable GC based on the results of the MAGIC [2] and FNCLCC/FFCD 9703 [3] trials that showed a significant improvement in overall survival (OS) for patients treated with preoperative CT (epirubicin, cisplatin, and 5-fluorouracil [ECF] in MAGIC trial and cisplatin, and 5-fluorouracil in FNCLCC/FFCD trial), as compared with surgery alone.

Docetaxel has been proven to exert significant activity in GC in several clinical trials and in different settings [4, 5], however classical DCF (docetaxel, cisplatin, and 5-fluorouracil) regimen was associated with high incidence of grade (G) 3–4 toxicities, including neutropenia in 82%, stomatitis in 21%, and diarrhea in 19% [4]. Docetaxel-based modified regimens as FLOT, consisting of 5-fluorouracil, leucovorin, oxaliplatin and docetaxel, have proved to be active in terms of complete pathological regression and to be more tolerable than classical DCF [6–8].

Recently, Al-Batran and colleagues [8, 9] showed that FLOT can be considered as a new standard of care in perioperative setting for locally advanced resectable gastric or gastro-oesophageal junction (GEJ) adenocarcinoma as it showed significant OS improvement compared to conventional ECF/ECX.

Despite these important findings, very few data concerning FLOT regimen in the real-world setting to date are available.

RealFLOT is an Italian, multicentric observational study, with the aim to describe the feasibility and activity in terms of pathological responses and survival outcomes of perioperative FLOT administered in routine clinical practice in patients with resectable gastric or GEJ cancer.

## Patients and methods

### Study population

This prospective observational study was approved by Institutional review board of Azienda Ospedaliero-Universitaria Careggi (Comitato Etico Regionale for clinical experimentation of Toscana region – Italy - Area Vasta Centro - 12818_oss) and by the review boards of all participating 15 Italian centers starting from September 2016. We obtained informed consent from each alive patient enrolled in the study. We collected clinical data from patients receiving perioperative FLOT treatment as established by routine clinical practice from September 2016 to September 2019.

The study population consisted of patients with histologically confirmed diagnosis of gastric or GEJ adenocarcinoma with clinical stage cT2 or higher and/or nodal involvement referring to the 7th edition of the International Union Against Cancer Tumour–Node–Metastasis classification, and suitable to underwent a R0-R1 resection, according to local surgical evaluation [10]. Clinical stage was assessed according to clinical local practice, routinely with CT scan. FDG-PET scan, US-endoscopy and diagnostic laparoscopy were not habitually used in clinical practice in all enrolling centers for staging.

Primary tumor location was classified according to Siewert for GEJ cancer [11]. Inclusion criteria also required at least 18 years of age and written informed consent. In contrast, patients with oesophageal cancer, squamous histology or metastatic disease at the time of initial diagnosis were excluded. All patients received at least one cycle of perioperative FLOT regimen, which consisted of 5-fluorouracil 2600 mg/m^2^ as 24-h infusion on day 1, leucovorin 200 mg/m^2^ on day 1, oxaliplatin 85 mg/m^2^ on day 1, and docetaxel 50 mg/m^2^ on day 1. All drugs were administered intravenously every 2 weeks. Dose modifications and delays, and treatment discontinuations were performed at the investigator’s discretion according to the observational nature of the study.

### Data collection and study endpoints

Clinical data including all available demographic information, medical history, diagnosis, chemotherapy, surgery including timing, type (total/subtotal gastrectomy and/or transhiatal/transthoracic esophagectomy), margin of resection (R0, R1, R2), lymphadenectomy (D1, D2, D3), pathological results, molecular analysis, microsatellite instability (MSI) status, clinical outcomes, adverse events (AEs), and laboratory alterations were collected from patients’ medical records. AEs were graded according to the National Cancer Institute Common Terminology Criteria for AEs version 4.03. Radiological response was assessed according to RECIST, version 1.1 [12]. It was also required to specify whether the pathological response was classified according to the Becker regression criteria [13].

The two primary endpoints were the evaluation of feasibility of perioperative FLOT and the assessment of pathological complete response (pCR) rate, in an Italian real-world patient population. pCR rate was defined as the proportion of patients with absence of residual tumor cells at pathological examination after preoperative CT.

The secondary endpoints were overall response rate (ORR), disease control rate (DCR), disease-free survival (DFS), and OS. Treatment adherence, safety, and correlation of clinical, biological and pathological factors with pathological response and survival outcomes were analyzed.

### Immunohistochemical staining of MMR proteins

Immunohistochemistry for the four most common mismatch repair proteins (MMR) MLH1,MSH2, MSH6 and PMS2 was evaluated at each enrolling center. Immunohistochemical staining was performed on formalin-fixed, paraffin-embedded tissue sections following manufacturer’s instructions according to local clinical practice and Italian Guidelines [14].

### Statistical analysis

Demographic and clinical data, safety and treatment exposure were analyzed using descriptive statistics. OS was defined as the time from the start of treatment to death from any cause. DFS was defined as the time from the start of treatment to disease progression, relapse, or death from any cause, whichever occurred first. Statistical comparisons for categorical variables were performed using *X*^2^ test. Time-to-event endpoints were estimated using the Kaplan–Meier method. Survival distributions for specific subgroups of patients were tested with log-rank test. Parameters with a statistically significant log-rank test were included in the multivariate Cox proportional hazard regression linear model to compare hazard ratio (HR) and 95% confidence interval (95% CI). A *p*-value of 0.05 or lower was considered to be statistically significant. Analyses were performed using Jamovi (www.jamovi.org) and R studio.

## Results

### Patient characteristics

Between September 2016 and September 2019, a total of 206 patients received perioperative FLOT regimen at 15 Italian centers: 141 patients (68%) were male and 65 (32%) were female. The median age was 63 years (range 36–77). The percentage of patients ≥65 years was 43%. Primary tumor locations (stomach and GEJ) were represented with similar rates (55 and 45%, respectively). All patients had a good clinical condition, with an ECOG performance status (PS) 0 or 1. The histological type on diagnostic biopsy was assessed by local pathologists. According to the Lauren classification, a diffuse type adenocarcinoma was reported in 13% of patients (*n* = 27), intestinal in 18.0% (*n* = 37) and mixed in 5.0% (*n* = 11). Unfortunately, Lauren subtype has not been specified in 64% of cases. The presence of signet ring cells was reported in 15.0% (*n* = 31) of cases.

At the time of initial diagnosis, clinical stage T3–4 was reported in 87% (*n* = 180) of patients and nodal involvement in 84% (*n* = 174). The baseline demographic and disease characteristics are shown in Table 1.

**Table 1:** Baseline demographic and disease characteristics of a cohort (*n* = 206) of resectable GC and GEJ adenocarcinoma patients treated with perioperative FLOT in a real-world setting

	Patients N (%)
**Sex**	
Male	141 (68%)
Female	65 (32%)
**Age (y)**	
Median (range)	63 (36–77)
< 65 years	117 (57%)
≥ 65 years	89 (43%)
**ECOG PS**	
0	189 (92%)
1	17 (8%)
**Location**	
Stomach	114 (55%)
GEJ	92 (45%)
**Lauren’s type**	
Intestinal	37 (18%)
Diffuse	27 (13%)
Mixed	11 (5%)
Not evaluable according to Lauren	134 (64%)
**Signet ring cells**	
Yes	31 (15%)
Not specified	175 (85%)
**cT**	
T1–2	26 (13%)
T3–4	180 (87%)
**cN**	
N0	32 (16%)
N1	61 (30%)
N2	73 (35%)
N3	40 (19%)
**Grading**	
G1	4 (2%)
G2	16 (8%)
G3	114 (55%)
Missing	72 (35%)

Abbreviations: *FLOT* fluorouracil, leucovorin, oxaliplatin, and docetaxel; *G* grade; *GEJ* gastro-oesophageal junction; *ECOG* Eastern Cooperative Oncology Group; *PS* performance status

Data about MSI status were available in 93 patients: 9 of those (9.7%) were MSI-H. In the MSI-H subgroup the median age was 72 years (range 57–75), 4 patients were female and 5 male. Primary tumor location was stomach in 89% and GEJ in 11%. At the time of the diagnosis, 6 patients had a stage III cancer, 2 patients had stage II, and one patient stage I (Table 2).

**Table 2:** Demographic and disease characteristics of MSI-H patients treated with perioperative FLOT in a real-world setting

MSI-H patient	Sex	Age at the time of diagnosis (years)	Primary tumor location	Lauren’s type	Baseline clinical stage	Clinical response
N1	Male	75	GC	NA	III	PR
N2	Female	65	GC	NA	III	PR
N3	Female	72	GC	Intestinal	III	SD
N4	Male	57	GC	Mixed	II	SD
N5	Male	73	GC	Diffuse	I	SD
N6	Male	73	GC	Intestinal	III	SD
N7	Male	69	GEJ	Intestinal	III	PD
N8	Female	73	GC	Diffuse	III	PR
N9	Female	67	GC	NA	II	SD

Abbreviations: *GC* gastric cancer; *GEJ* gastro-oesophageal junction; *MSI-H* microsatellite instability-high; *NA* not assessed; *PD* progressive disease; *PR* partial response; *SD* stable disease

### Treatment exposure

**Fig. 1:**
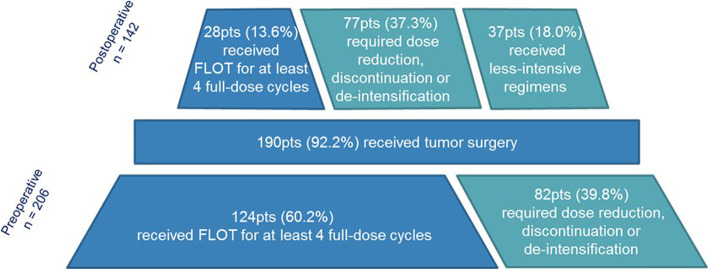
Treatment exposure. Percentages refer to the total study population (*n* = 206). Abbreviations: FLOT: fluorouracil, leucovorin, oxaliplatin, and docetaxel; pts.: patients

Treatment exposure data are shown in Fig. 1. In the preoperative phase, 186 out of 206 patients (90.3%) received at least 4 preoperative cycles of CT regardless of dose reduction or de-intensification, with a median number of cycles of 4 (range 1–8). One hundred twenty-four patients (60.2%) received FLOT for at least 4 full-dose cycles. Dose reduction, discontinuation or de-intensification were required for the remaining 82 patients (39.8%). Treatment discontinuation occurred in 5.8% (*n* = 12) of patients in the preoperative phase, mainly due to unacceptable toxicity.

A total of 142 patients (68.9%) started the postoperative phase. Among these patients, 105 (51.0% of the study population) received FLOT and 37 (18%) received less intensive regimens (e.g. mFOLFOX6 [5-fluorouracil, leucovorin, and oxaliplatin], infusive 5FU/leucovorin or capecitabine). The main reasons for administering a less intensive treatment regimen or not administering the postoperative phase at all were due to the clinical conditions after surgery and/or toxicities experienced in the preoperative phase.

Eight patients received a radiation therapy, 3 of whom after R1 resection. The median number of postoperative cycles of any regimen was 4 (range 1–11). Overall, only 28 patients (13.6%) received FLOT for at least 4 full-dose cycles in the postoperative phase. In contrast, 77 patients (37.3%) required dose reduction, treatment discontinuation or de-intensification. Among patients who started the postoperative phase, a treatment discontinuation of 12.0% was observed. At the time of this analysis, 4 patients were still under postoperative treatment.

### Safety

Grade 3–4 AEs were reported in Table 3. Neutropenia was the most commonly reported G 3–4 AE both in the preoperative and postoperative phase, with an incidence of 19.9 and 16.9%, respectively. However, febrile neutropenia was experienced by only 4.4% of patients in the preoperative phase and 1.4% of patients in the postoperative phase. Granulocyte-colony stimulating factors (G-CSFs) were administered as primary or secondary prophylaxis in 69.0% of patients in the preoperative phase and in 58.4% of patients who started the postoperative phase.

**Table 3:** Grade 3–4 adverse events

	RealFLOT: Preoperative (***n*** = 206)	RealFLOT: Postoperative (***n*** = 142^a^)	Patients ≥ 65 in the preoperative phase (***n*** = 89)	Patients < 65 in the preoperative phase (***n*** = 117)	Patients ≥ 65 in the postoperative phase (***n*** = 56^a^)	Patients < 65 in the postoperative phase (***n*** = 86^a^)
**Hematologic AEs**						
Neutropenia	19.9%	16.9%	18.0%	21.4%	14.3%	18.6%
Febrile neutropenia	4.4%	1.4%	4.5%	4.3%	1.8%	1.2%
Anemia	0.5%	0.7%	0%	0.8%	0%	1.2%
**Gastrointestinal AEs**						
Diarrhea	1.5%	3.5%	2.2%	0.8%	3.6%	3.4%
Stomatitis	0.5%	1.4%	1.1%	0%	1.8%	1.2%
Nausea	2.4%	5.6%	3.4%	1.7%	1.8%	8.1%
Vomiting	1.9%	3.5%	4.5%	0.8%	1.8%	4.6%
Increases in ALT/AST	0.5%	0%	0%	0.8%	0%	0%
Infective events (any G)	5.8%	3.5%	5.6%	6.0%	5.4%	2.3%
Neurotoxic effects	1%	2.8%	2.2%	0%	1.8%	3.5%
Cardiac complications	0.5%	0%	1.1%	0%	0%	0%
Thromboembolic events (any G)	2.4%	1.4%	0%	4.3%	1.8%	1.2%

^a^ At the time of the interim analysis, 4 patients were still under postoperative treatment

Abbreviations: *AE* adverse event; *G* grade

The frequency of gastrointestinal G 3–4 AEs was higher in the postoperative phase than in the preoperative phase (14.0 and 6.5%), including nausea in 5.6 and 2.4%, diarrhea in 3.5 and 1.5%, and stomatitis in 1.5 and 0.5%, respectively. Moreover, a non-negligible incidence of other G 3–4 AEs was reported, including infectious complications in 5.8 and 3.5% and thromboembolic events in 2.4 and 1.4%, respectively. Notably, a low incidence of G 3–4 peripheral neuropathy (1 and 2.8%, respectively) was observed. At the time of the last follow-up, however, G1 neurotoxicity was reported in 15% of patients, G2 in 2.9%, and G3 in 1.0%.

Mortality rates at 30, 60, and 90 days for resected patients were 1.0, 2.1, and 4.7%, respectively; unfortunately, the comprehensive data regarding surgical morbidity were unavailable.

No differences were registered between patients aged < 65 and ≥ 65 years in terms of perioperative mortality, while a trend towards a higher incidence of G ≥ 3 AEs affecting the elderly was observed in preoperative phase (*p* = 0,014), especially when considering gastrointestinal toxicities (*p* < 0,00001).

### Surgical and pathological findings

A total of 191 out of 206 patients (92.7%) had surgical resection. One of these patients was lost to follow-up after surgery and it was not possible to recover the clinical-pathological details; therefore, subsequent analyses considered 190 operated patients (92.0%) whose clinical information were available.

The main reasons for not proceeding to radical surgery were the detection of metastatic disease at exploratory laparoscopy in 3 patients, disease progression in 9, and not specified in 3.

Surgical and pathology results are shown in Table S1 (Supplementary). Of 190 patients undergoing surgery, 81 underwent a total gastrectomy (42.6%) and 52 a subtotal gastrectomy (26.4%). Of the remaining patients (30.0%) who underwent esophagectomy, the majority received a transthoracic procedure (17.4%). The vast majority of operated patients underwent D2 lymphadenectomy (82.1%) and R0 resection (92.1%). Surgery was performed 4.5 months after diagnosis (median), while the median time from end of neoadjuvant CT to surgery and from surgery to start of adjuvant CT was 0.5 and 1.0 month, respectively. Pathological stage yT0 was obtained in 12.1% of cases and pathologic node-negativity (ypN0) in 31.6%.

In the study population, 15 patients (7.3%) achieved a pCR. In addition, 28 patients (14.7%) achieved a pathological stage I according to the eight edition of the American Joint Committee on Cancer [15]; while pathological stage II, III and IV resulted in 75 (39.5%), 61 (32.1%), and 11 patients (5.8%), respectively. ORR and DCR were 45.6 and 94.2%, respectively.

Among patients who underwent radical surgery, a pathological downstaging, defined as pathologic stage less than clinical stage, was obtained in 45.5% of cases.

### Survival outcomes

At the time of this analysis, 48 of 206 patients (23.3%) died and 3 were lost to follow-up. Seventy-one patients (34.5%) had a rapid disease progression during preoperative phase, relapse after surgery or death. Among patients who had a resective surgery, 3 (1.6%) experienced a local recurrence, 45 (23.7%) distant recurrence, and 8 (4.2%) both local and distant recurrence.

**Fig. 2:**
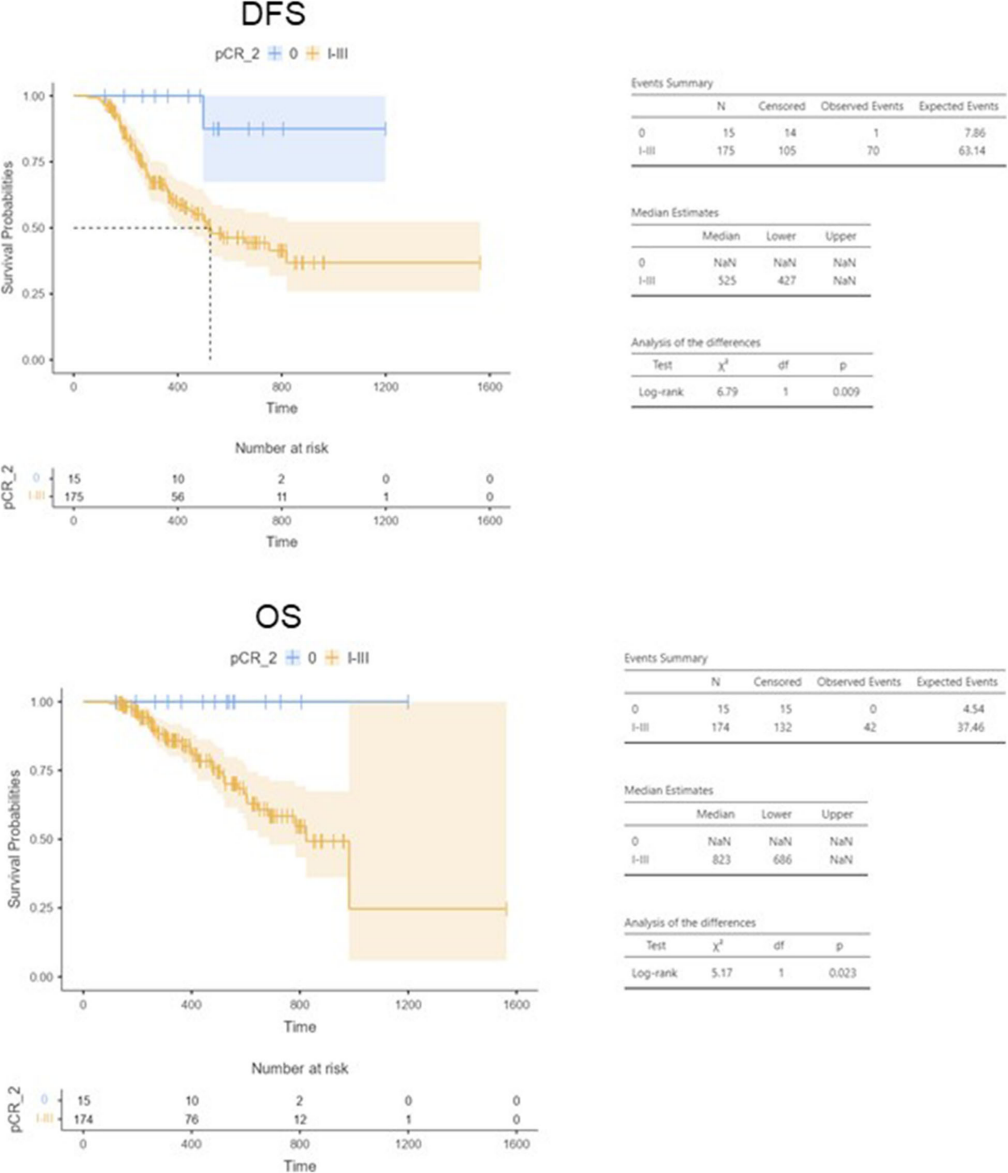
Disease free survival (DFS) and overall survival (OS). Abbreviations: DFS: disease-free survival; OS: overall survival; pCR: pathological complete response; NaN: not reached

With a median follow-up of 11.9 months, data on OS and DFS were to date immature. However,

both DFS and OS were significantly longer in patients achieving a pCR (DFS: 17.2 months [95% CI 14.0 - NaN] vs NaN, *p* = 0.009; OS: median 27.0 months [95% CI 22.5 – NaN] vs NaN, *p* = 0.023) (Fig. 2) compared with those achieving a non-complete response. Significant differences were observed in DFS and OS also in patients with a pathological stage 0-I than II-IV (DFS *p* < 0.001, OS *p* < 0.001), and 0-II than III-IV (DFS *p* < 0.001, OS *p* = 0.003).

In addition, DFS was significantly longer in patients with ypT0–2 than ypT3–4 (p = 0.003), and ypN0 than ypN+ (*p* < 0.001). In contrast, there were no significant differences according to primary tumor location (*p* = 0.991), presence/absence of signet ring cell (*p* = 0.709), age (< 65 years vs ≥65 years, *p* = 0.890) and starting postoperative phase with FLOT or less intensive regimens (*p* = 0.071).

**Fig. 3:**
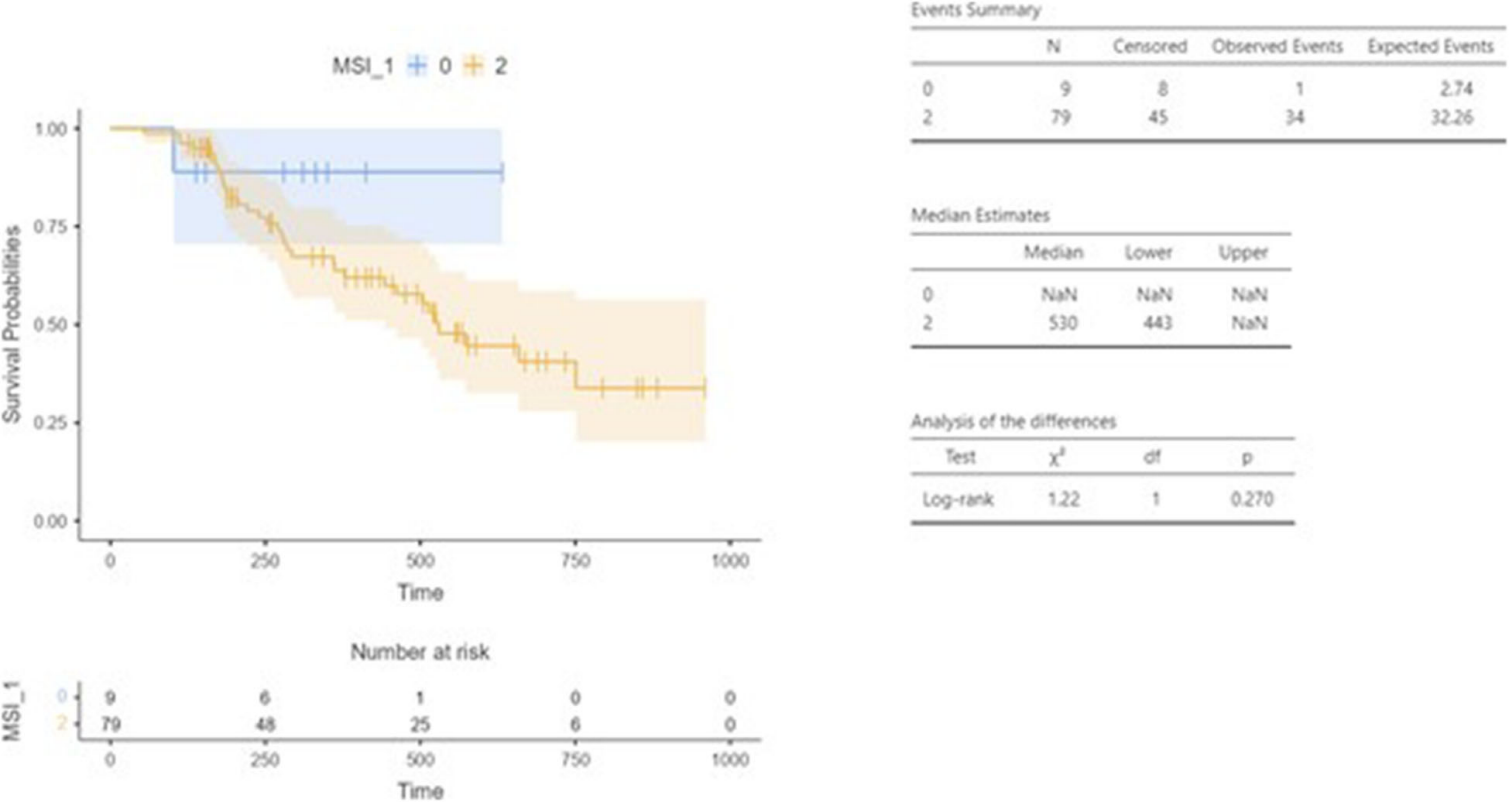
Disease free survival (DFS) according to MSI status. 0 = MSI-H; 2 = MSS

For MSS patients DFS was 17.4 months, whereas median DFS was not reached for MSI-H patients (95% CI 14.5 - NaN vs NaN, *p* = 0.270) (Fig. 3).

Univariate analysis showed that ypT ≥ 2, ypN ≥ 2, intraoperative detection of metastasis and pathological stage ≥3 were negatively associated with DFS, while postoperative CT was positively associated (Table 4). Multivariate analysis confirmed the association between DFS and pathological stage ≥3, intraoperative detection of metastasis, and postoperative CT (Table 4).

**Table 4:** Univariate and multivariate analysis for DFS

	UNIVARIATE		MULTIVARIATE	
Variable	HR with 95% CI	***p*** value	HR with 95% CI	***p*** value
Pathological stage				
1	1.3 (0.12–14)	*0.8500*	0.48 (0.02–8.74)	*0.616*
2	7 (0.95–52)	*0.0560*	5.79 (0.43–78.08)	*0.185*
3	14 (1.9–100)	***0.0093***	30.36 (1.75–526)	***0.019***
4	30 (3.8–230)	***0.0013***	–	*–*
ypT1	2.6 (0.63–11)	*0.1800*	4.8 (0.72–32.05)	*0.104*
ypT2	3.6 (1.1–12)	***0.0410***	1.76 (0.36–8.68)	*0.487*
ypT3	5.6 (1.7–18)	***0.0043***	0.95 (0.20–4.37)	*0.943*
ypT4	4.7 (1.2–18)	***0.0250***	0.79 (0.15–4.19)	*0.784*
ypN1	1.9 (0.85–4.4)	*0.1100*	0.68 (0.23–2.03)	*0.487*
ypN2	3.3 (1.5–7.3)	***3.2e-03***	0.55 (0.16–1.89)	*0.343*
ypN3	4.8 (2.3–10)	***4.6e-05***	0.39 (0.09–1-54)	*0.178*
IntraOP MTS	3.9 (2–7.8)	***7.6e-05***	56.8 (3.5–906)	***0.004***
Postoperative CT	0.33 (0.2–0.54)	***9.3e-06***	0.32 (0.19–0.54)	***2.3 e-05***

Abbreviations: *CT* chemotherapy; *IntraOP MTS* intraoperative metastasis; *ypN* postoperative N stage after preoperative chemotherapy; *ypT* postoperative T stage after preoperative chemotherapy

As regards OS, univariate analysis showed that ypN ≥ 2 and intraoperative detection of metastasis were negatively associated, while postoperative CT was positively associated (Table 5). Postoperative CT was associated with better OS in multivariate analysis and, on the contrary, ypN ≥ 2 was associated with poor survival (Table 5).

**Table 5:** Univariate and multivariate analysis for OS

	UNIVARIATE		MULTIVARIATE	
Variable	HR with 95% CI	***p*** value	HR with 95% CI	***p*** value
ypN1	2.3 (0.77–6.7)	0.14	2.65 (0.88–8.02=	0.084
ypN2	2.8 (0.91–8.4)	0.07	3.19 (1.03–9.97)	**0.044**
ypN3	5.5 (2–15)	**9.6e-4**	4.95 (1.73–14.11)	**0.003**
IntraOP MTS	2.7 (1–7)	**0.039**	1.84 (0.68–4.96)	0.23
Postoperative CT	0.36 (0.19–0.67)	**0.0014**	0.35 (0.18–0.66)	**0.0012**

Abbreviations: *CT* chemotherapy; *IntraOP MTS* intraoperative metastasis; *ypN* postoperative N stage after preoperative chemotherapy

### Statistical comparisons

The analysis of clinicopathological factors associated with a lower pathological stage is reported in Table S2. Pathological stage was associated with clinical T stage (*X*^2^ 34.20; *p* < 0.001), clinical N stage (*X*^2^ 38.61; *p* < 0.001), grading (*X*^2^ 17.09; *p* = 0.029). In contrast, there were no significant differences according to age (*p* = 0.473), sex (*p* = 0.326), ECOG PS at baseline (*p* = 0.805), primary tumor location (*p* = 0.324), at least 4 full-dose preoperative cycles (*p* = 0.067), gastrointestinal G 3–4 AEs (*p* = 0.759), hematological G 3–4 AEs (*p* = 0.622), toxicity-induced dose delay or treatment discontinuation (*p* = 0.104), type of surgery (*p* = 0.689), type of lymphadenectomy (*p* = 0.515), human epidermal growth factor receptor 2 (HER2) status (*p* = 0.800), and Lauren histotype (*p* = 0.059).

## Discussion

Since the publication of the FLOT4-AIO study, perioperative FLOT is considered the new standard of care in patients with resectable GC or GEJ adenocarcinoma [8, 9]. To date, no large studies have been carried out to assess the safety and efficacy of this regimen in the routine clinical practice. As known, data reported outside clinical trials are crucial to understand whether the results can be transferable to a real-world setting. To this aim, the RealFLOT study described the use of perioperative FLOT in 206 patients treated at 15 Italian centres.

In our study the primary endpoint was pCR, which was obtained in 7.3% of the overall population enrolled. Such result was lower than expected, considering the pCR rates of the FLOT regimen reported in FLOT4-AIO (16%), in NeoFLOT (20%) and in an observational small trial conducted by Homann et al. (17.4%) [6–9].

The pathological regression rate registered, however, was obtained in a non-favourably selected population with a more advanced clinical stage at the time of diagnosis. In fact, cT3–4 and cN positive stage in our study were reported in 87 and 85% respectively, while were 83 and 78% in the FLOT4-AIO trial. Moreover, the treatment exposure in our study was lower than expected. In particular, 39.8% of patients required dose reductions, less intensive regimens (e.g FOLFOX or single-agent 5-FU) or treatment discontinuation in the preoperative phase. Conversely, the rate of patients who required dose modification in the FLOT4-AIO study was roughly the half (19%) during preoperative phase [8, 9].

Pathological response has been reported to be different according to Lauren histotype [6–8]. Although Lauren histotype was assessed in diagnostic specimens only in 36% of patients, our data confirm a trend towards a lower pathological stage after CT for the intestinal subtype respect to diffuse one.

As previously reported [7, 8, 16, 17], we confirm pCR as a predictive parameter of survival. Although the survival data at the time of this analysis were still immature, DFS and OS were significantly longer (*p* = 0.009 and *p* = 0.023, respectively) in patients who achieved a pCR respect to other type of responses. In addition, in our study, ypT and ypN were significantly associated with DFS (*p* = 0.003 and *p* < 0.001, respectively). This was consistent with the retrospective study of Schmidt et al. in which ypT and ypN were identified as independent prognostic factors on multivariate analysis [18].

Overall, 142 patients (68.9%) started a postoperative treatment including 105 patients (51%) who received the triplet. Notably, only 28 patients (13.6%) received FLOT for at least 4 full-dose cycles.

As expected, postoperative toxicities were significantly higher than preoperative ones, particularly for gastrointestinal G 3–4 AEs.

Although different approaches, including neoadjuvant CT and chemoradiotherapy (CTRT), have been studied in phase III trials, the best treatment strategy for resectable patients worldwide is still debated, in particular for GEJ subgroup [9, 19]. These real-word data corroborate that in Italy, to date, perioperative FLOT is regarded as the treatment of choice in patients with ECOG PS 0–1, as demonstrated by the relevant accrual.

Safety profile was consistent with literature. Overall, neutropenia was the most commonly reported G 3–4 AE, affecting 41 patients out of 206 (19.9%) in the preoperative phase and 24 out of 142 (16.9%) in the postoperative phase. Notably, G-CSF were widely administered (69.0 and 58.4% of patients in the preoperative and postoperative phase, respectively) in order to maintain dose intensity and this could partly explain (together with schedule modifications) the lower incidence of G 3–4 neutropenia compared with FLOT4. In addition, no toxic death was observed and 30-day postoperative mortality rate was 1.0%.

Furthermore, FLOT regimen seems to be feasible and effective also in elderly patients. Among 89 patients (43%) aged 65 or older, the safety profile was similar to younger patients, although gastrointestinal and hematologic toxicities deserve a special attention in this population. In addition, the distribution of pathological stage and DFS did not differ according to age group.

Although the positive association between MSI-H and better prognosis in localized GC has been reported in several studies [20, 21], the evidence for MSI being a negative predictive factor of the efficacy of CT in neoadjuvant or adjuvant setting is not definitively confirmed [22, 23] . In addition, taxane-based regimens were not included in published meta-analysis by Pietrantonio et al. and the role of MSI status in patients treated with taxane-containing triplets is poorly explored in literature [21–24].

In Table 2 we detailed the characteristics of MSI-H patients subgroup treated with perioperative FLOT in our study, including the assessment of clinical response and we noticed that, consistently with literature, primary tumor location was predominantly stomach (89%) respect to GEJ (11%). Regarding to clinical response rate, ORR was 33% in this subgroup, inferior to those observed in entire population.

In our survival analysis, the MSI-H tumors had better DFS than MSS ones, confirming previous published data [24]. Although limited in sample size and with a still immature follow-up, our study supports the association of MSI status with survival parameters in a real-life setting. Therefore, MSI-H status is suggested to be a positive prognostic marker also in patients treated with a taxane-containing triplet (FLOT) in the perioperative setting. In this small subset of patients, considering the data yet published on the potential worst response to treatment respect to MSS group, the possibility of immediate surgery instead of CT treatment could be considered [22]. However, it will be crucial to wait for ongoing analyses from prospective trials before definitely validating MSI as a predictor of the efficacy of systemic treatment with taxane-based CT.

Due to observational nature of study, there are obviously some limitations. The analysis of histological samples was not centrally assessed by a pathologist blinded to the clinicopathological characteristics. The pathological regression was assessed according to the Becker criteria only in 26.8% of patients. The low rate of histotype assessment on diagnostic biopsy precluded a precise estimate of the differences in pathological response. Finally, according to the observational nature of the study, there was a notable difference in the assessment of clinical stage, specifically for the use of endoscopic ultrasound, laparoscopy, and PET-FDG. In addition, MSI status was available in only 93 patients, and it was generally performed on surgical specimen after treatment, so we have no data about MSI status of patients who achieved a pCR.

Several clinical trials are currently evaluating perioperative FLOT in combination with other agents in order to improve pCR rate and survival outcomes. In a phase II trial (PETRARCA, NCT02581462) FLOT in combination with trastuzumab and pertuzumab was associated with high rates of PCR and node negativity in HER2-positive esophagogastric adenocarcinoma [25]. Additionally, the combination of FLOT with pembrolizumab is under investigation in the phase III KEYNOTE-585 trial (NCT03221426).

## Conclusions

Although this study exhibits some limitations, Italian RealFLOT experience is the first large real-word analyses that confirmed that perioperative FLOT is feasible and safe in good PS patients with resectable GC or GEJ adenocarcinoma in clinical daily practice. The study also confirmed pCR as the most important predictive markers of survival. In addition, data suggests MSI-H status as a potential positive prognostic factor. Strategies to improve treatment exposure, particularly in the postoperative phase, are warranted.

## Supplementary Information

**Additional file 1.**

## Data Availability

All data generated or analyzed during this study are included in this published article.

## Abbreviations

AE: Adverse event
CT: Chemotherapy
CTRT: Chemoradiotherapy
DCF: Docetaxel, cisplatin, 5-fluorouracil
DCR: Disease control rate
DFS: Disease-free survival
ECF: Epirubicin, cisplatin, 5-fluorouracil
FLOT: Fluorouracil, leucovorin, oxaliplatin, and docetaxel
FOLFOX: Fluorouracil, leucovorin, and oxaliplatin
G: Grade
GC: Gastric cancer
GEJ: Gastro-oesophageal junction
G-CSF: Granulocyte-colony stimulating factor
HER2: Human epidermal growth factor receptor 2
MSI: Microsatellite instability
MSS: Microsatellite stable
ORR: Overall response rate
OS: Overall survival
pCR: Pathological complete response
PS: Performance status
MMR: MLH1,MSH2, MSH6 and PMS2
