# Supplementary material for: Results of the observational prospective RealFLOT study
Source: BMC Cancer. 2021 Oct 8;21:1086. doi: 10.1186/s12885-021-08768-7 (PMC8499559; doi:10.1186/s12885-021-08768-7)
Supplement: Supplementary file 1 — Additional file 1. [file 12885_2021_8768_MOESM1_ESM.docx]

**Results of the observational prospective RealFLOT study**

E. Giommoni, D. Lavacchi, G. Tirino, L. Fornaro, F. Iachetta, C. Pozzo, M.A. Satolli, A. Spallanzani, M. Puzzoni, S. Stragliotto, M. Sisani, V. Formica, F. Giovanardi, A. Strippoli, M. Prisciandaro, S. Di Donato, L. Pompella, I. Pecora, A. Romagnani, S. Fancelli, M. Brugia, S. Pillozzi, F. De Vita, L. Antonuzzo.

**Supplementary materials**

**Table S1**. Surgical and pathological findings of operated GC and GEJ adenocarcinoma patients (n= 190) in the RealFLOT study.

|  | **Resected patients**  **N (%)** |
| --- | --- |
| **Type of surgery** | |
| Total gastrectomy  Subtotal gastrectomy  Transthoracic gastrectomy  Other surgery | 81 (42.6%)  52 (27.4%)  33 (17.4%)  24 (12.6%) |
| **Type of lymphadenectomy** | |
| D1  D2  D3  Others | 8 (4.2%)  156 (82.1%)  21 (11.1%)  5 (2.6%) |
| **Resection grade** | |
| R0  R1  R2 | 175 (92.1%)  14 (7.4%)  1 (0.5) |
| **ypT** | |
| T0  T1-2  T3-4 | 23 (12.1%)  74 (38.9%)  93 (48.9%) |
| **ypN** | |
| N0  N1  N2  N3 | 60 (31.6%)  44 (23.1%)  38 (20.0%)  48 (25.3%) |

Abbreviations: GC: gastric cancer; GEJ: gastro-oesophageal junction; ypN: postoperative N stage after preoperative chemotherapy; ypT: postoperative T stage after preoperative chemotherapy.

**Table S2.** Contingency analysis (*X*^2^ test)

| **Variables** | | ***X*^2^** | **P** |
| --- | --- | --- | --- |
| cT | Pathological stage | 34.20 | <0.001 |
| cN | Pathological stage | 38.6 | <0.001 |
| ECOG PS at baseline | Pathological stage | 1.62 | 0.805 |
| Sex | Pathological stage | 4.65 | 0.326 |
| Age | Pathological stage | 3.53 | 0.473 |
| Primary tumor location | Pathological stage | 4.66 | 0.324 |
| At least 4 full-dose cycles | Pathological stage | 8.78 | 0.067 |
| Gastrointestinal G3-4 AEs | Pathological stage | 1.87 | 0.759 |
| Hematologic G3-4 AEs | Pathological stage | 2.63 | 0.622 |
| Toxicity-induced dose delay or treatment discontinuation | Pathological stage | 7.68 | 0.104 |
| Type of surgery | Pathological stage | 5.63 | 0.689 |
| Type of lymphadenectomy | Pathological stage | 7.20 | 0.515 |
| HER2 | Pathological stage | 1.65 | 0.800 |
| Grading | Pathological stage | 17.09 | 0.029 |
| Lauren histotype | Pathological stage | 18.09 | 0.059 |

Abbreviations: AE: adverse event; cN: clinical N stage; cT: clinical T stage; G: grade; HER2: human epidermal growth factor receptor 2; PS: performance status.
